# Himplant^®^ subcutaneous penile implant improves penile appearance and erectile dysfunction after radical prostatectomy: a case series

**DOI:** 10.1038/s41443-024-00857-y

**Published:** 2024-03-05

**Authors:** Robert J. Valenzuela, James J. Elist, Daniel Moon, Luka Cvijanovic, Steven K. Wilson

**Affiliations:** 1 Department of Urology, Icahn School of Medicine at Mount Sinai, New York, NY USA; 2 Emeritus, Department of Urology, Cedars-Sinai Medical Center, Los Angeles, CA USA; 3 James J. Elist M.D., A Medical Corporation, Beverly Hills, CA USA; 4 Institute for Urologic Excellence, La Quinta, CA USA

**Keywords:** Erectile dysfunction, Quality of life, Prostatic diseases

## Abstract

Erectile dysfunction is a major postoperative complication following radical prostatectomy. Various treatments for post- radical prostatectomy erectile dysfunction including nonsurgical phosphodiesterase-5 inhibitors, intraurethral alprostadil, intracavernosal injections and penile implant prosthesis, often yield suboptimal results. In this prospective single-center case series, we examine the efficacy and outcomes of Himplant^®^, a subcutaneous silicone penile implant, placement in four patients with post-radical prostatectomy erectile dysfunction who experienced limited benefits with phosphodiesterase-5 inhibitors. Patient data including demographics, prostate cancer diagnoses, erectile dysfunction characteristics, previous treatments, and outcomes were collected. Himplant^®^ placement was performed in a standardized manner through a high scrotal incision in all cases. Follow-up evaluations were conducted to assess the effectiveness of the procedure and any associated complications. Patients were contacted and asked 15 questions regarding satisfaction and erectile function with the responses recorded. This study presents findings of high patient satisfaction, increases in flaccid penile length and girth, no incidence of adverse events, and improved erectile function following Himplant^®^ placement post-radical prostatectomy. Accordingly, we suggest Himplant^®^ placement in patients who are frustrated by their penile appearance and suffering from erectile dysfunction after radical prostatectomy. Further multicenter studies are warranted to validate these findings and assess long-term outcomes and patient-reported satisfaction.

## Introduction

Erectile dysfunction (ED) is a common and known complication of radical prostatectomy (RP), with prevalence rates ranging from 14% to 90% [1, 2]. Post-RP ED can result from injury of the cavernous nerves [3], neuropraxia [4], and/or incomplete nerve-sparing surgery [5]. The increased prevalence and early detection of prostate cancer in modern times have contributed to a higher proportion of young RP cases, highlighting the need for post-RP ED treatment. In addition to discontent with ED, many men are also bothered by the appearance of a shortened [6], shrunken, and/or thin penis following RP [7].

For patients with ED who are irresponsive to, reject, or have contraindications to less-invasive therapies such as oral phosphodiesterase-5 inhibitors (PDE-5i) and intracavernosal injections, penile prosthesis surgery represents an excellent treatment option [8–10]. Penile rigidity, ability to achieve penetration, intercourse frequency, and patient satisfaction were superior in patients who had undergone penile implantation for post-RP ED compared to those who were on PDE-5i alone [11, 12]. Two main types of penile prostheses are presently on the market: inflatable penile prosthesis (IPP) and malleable penile prosthesis (MPP). Despite being the gold standard in terms of high satisfaction and low complication rates, IPP has been associated with urethral injury and loss of penile length [13–15] while MPP has been associated with penile skin perforation, erosion, and implant fracture [16–18].

Himplant^®^ (International Medical Devices Inc., Beverly Hills, CA, USA), previously known as Penuma^®^, is a medical-grade solid silicone penile implant that has been granted four 510(k) clearances by the U.S. Food and Drug Administration (FDA), with indications that include cosmetic correction of penile soft tissue deformities [19] and cosmetic enhancement of the penis [20]. It is designed primarily for healthy men seeking to augment flaccid and erect girth, as well as to enhance the perceived length of the flaccid penis [21]. Furthermore, the Himplant^®^ provides axial rigidity, which supports penetrative sexual activity, making it a potentially advantageous option for patients with ED. In this study, we present four recent cases of Himplant^®^ placement post-RP to provide penile enhancement, with the added benefit of improved erectile function.

## Subjects and methods

This is a single-center case series discussing the outcomes of Himplant^®^ placement in four patients with post-RP ED who sought cosmetic enhancement of the penis. The study enrolled circumcised men aged between 18 and 65 years old who perceived an inadequate penile girth or length. Suitable candidates included those with a retractile penis, a reduction in penile size post-RP or other trauma, and congenital or acquired mild curvature of the penis (less than 30 degrees) without concurrent indentation deformities. Patients were also eligible if they expressed personal perceptions of insufficient penile size.

Exclusion criteria were strict to ensure patient safety and the integrity of the study results. Men unable to provide informed consent, those with an uncircumcised penis, a micro-penis, a history of penile girth enhancement, previous Xiaflex^®^ (Auxilium Pharmaceuticals Inc., Chesterbrook, PA, USA) injection, immunosuppression including HIV, or those currently on non-interruptible anticoagulant medication were not considered. Additional exclusion factors included uncontrolled diabetes, active genitourinary skin infection, a history of recurrent or active urinary tract infection, non-compliance with pre- or post-operative instructions, and smoking habits that could not be ceased 2 weeks pre- and at least 6 weeks post-surgery as required.

All four of our patients initially sought out the Himplant^®^ procedure to augment their penis. As a group, they were all dissatisfied with the appearance of their penis post-RP. Upon consultation, all patients declined or had previously declined IPP placement as a treatment option. Written and verbal consent was obtained from all patients, and they were informed of the possible risks, complications, benefits, and alternatives, such as no surgery or an IPP. Institutional Review Board (IRB) approval for reporting all outcomes associated with Penuma^®^/Himplant^®^ procedures was obtained and updated on April 18, 2023 [22]. The patients signed an informed consent form in agreement with the publication of this study and its accompanying images. All patients chose to undergo Himplant^®^ placement which was performed between May 2019 and November 2022.

### Himplant^®^ specifications

**Fig. 1:**
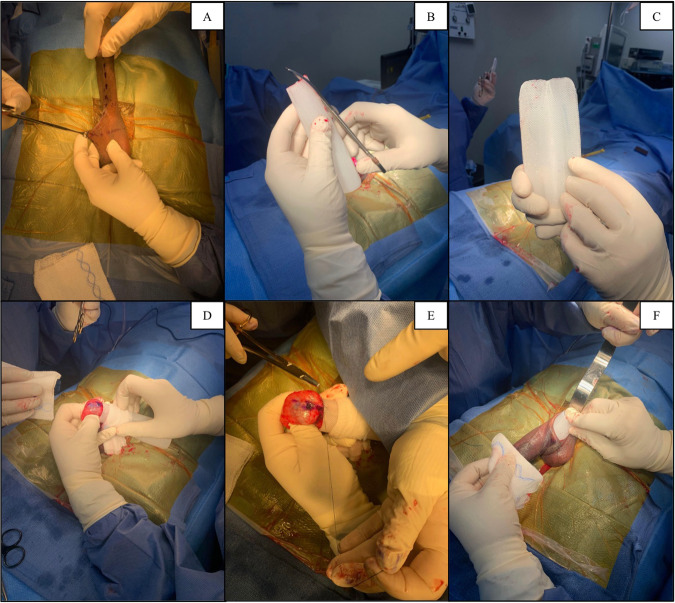
Himplant® surgery. **A** Scrotal incision. **B** Himplant® pre-trim. **C** Himplant® post-trim. **D** Himplant® placement. **E** Himplant® suture placement. **F** Himplant® insertion.

The Himplant^®^ is a penile prosthesis made of medical-grade silicone that is implanted subcutaneously through a high scrotal incision along the penile shaft [23]. Its wall thickness ranges longitudinally from 1.5 to 2.5 cm, and it is offered in three lengths: 14, 16, and 18 cm. All four patients in this study received the 16 cm Himplant^®^ (Fig. 1).

### Case 1

A 60-year-old man with a history of prostate cancer underwent RP in 2009 and presented to our clinic in 2019 for penile enhancement. He presented with complaints of refractory ED and penile shortening following RP. Initially managing ED with daily sildenafil (20 mg), his erectile function progressively worsened, leading to increased medication doses up to 100 mg with no success. Upon examination, this patient was diagnosed with moderate ED and severe penile retraction secondary to RP. This patient was offered IPP due to refractory ED but opted for Himplant^®^ instead and underwent surgery on May 13, 2019. Postoperatively, he resumed sexual activity within two months, achieving satisfactory erectile tumescence without medications by five months. At his 2023 follow-up, he reported continued satisfaction and no complications.

### Case 2

**Fig. 2:**
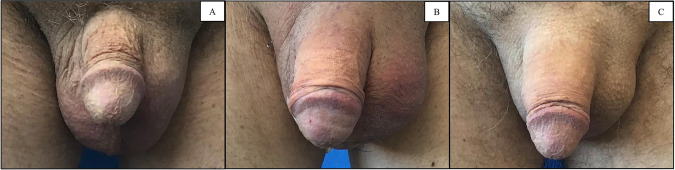
[Case 2] Pre- and postoperative images. **A** Pre-op front view of the flaccid penis. **B** 3-day post-op front view of the flaccid penis. **C** 12-month post-op front view of the flaccid penis.

A 64-year-old patient with a history of diabetes, hypogonadism, hyperlipidemia, tonsil cancer and prostate cancer, sought penile enhancement at our clinic in 2021 due to severe penile retraction (Fig. 2A). He underwent RP in 2006 and reported subsequently developing refractory ED. He used tadalafil (5 mg and 10 mg) but discontinued it due to an unsuccessful trial. He denied injection therapy due to trypanophobia. Upon examination, this patient was diagnosed with mild ED and penile retraction secondary to RP. This patient declined the option for IPP and underwent Himplant^®^ placement on February 9, 2021 (Fig. 2B). He reported improved erectile function immediately after the two months restriction period, achieving spontaneous erections and satisfactory erectile tumescence without medication. Upon follow up in 2023, he continued to utilize the implant satisfactorily without any complications (Fig. 2C).

### Case 3

A 56-year-old patient with a history of prostate cancer underwent RP in 2019 and presented to our clinic in 2021 seeking penile enhancement. He presented with complaints of refractory ED, severe penile retraction, and penile shortening. He used sildenafil (20 mg) and tadalafil (20 mg) to augment his erections but discontinued both due to minimal effects. Upon examination, this patient was diagnosed with moderate ED, penile shortening, and severe penile retraction secondary to RP. He declined IPP and opted for Himplant^®^ instead which was performed on September 23, 2021. Postoperatively, he resumed sexual activity within two months. Immediately thereafter, the patient confirmed satisfactory erectile tumescence and the ability to achieve and maintain erections naturally without medications. At the 2023 follow-up, he reported continued satisfaction with the device and no long-term sequelae.

### Case 4

A 65-year-old male with a history of hypertension, hypogonadism, polycythemia, and prostate cancer presented to our clinic in 2022 seeking penile enhancement. He underwent RP in 2017 for prostate cancer with subsequent chemo- and radiation therapy. After surgery, the patient reported refractory ED and a smaller, thinner penis. Upon examination, this patient was diagnosed with moderate ED, penile retraction, and penile narrowing secondary to RP. Despite limited success with tadalafil (20 mg and 40 mg) and Trimix injections, he declined IPP and opted for Himplant^®^ which was performed on November 15, 2022. Two months after surgery, the patient was permitted to engage in penetrative sex. After his first sexual encounter following Himplant^®^ surgery, the patient revealed that he was able to penetrate without erection or tumescence, due to the firmness of the implant. Importantly, he reported being able to achieve and maintain erectile tumescence following penetrative stimulation. Upon follow-up in 2023, the patient was pleased with both his erectile function and the appearance of his penis. He had no issues or long-term sequelae.

After surgery, patient follow-up was conducted on a weekly basis for the initial two months and monthly thereafter for 13–55 months. Patients were called and asked to complete the International Index of Erectile Function (IIEF), a validated questionnaire consisting of 15 questions across five domains: erectile function, orgasmic function, sexual desire, intercourse satisfaction, and overall satisfaction [24]. Informed consent was obtained in writing from all patients before undergoing the procedure.

## Results

All four patients experienced measured increases in flaccid dorsal length and flaccid midshaft girth (Table 1). The data is normally distributed. Mean increases in flaccid dorsal length and midshaft girth equaled 4.375 cm and 3.595 cm, respectively, with these changes being statistically significant (*p* = 0.005). While Cohen’s d effect size was greater than 3, indicating a large effect, the statistical power of the study was moderate. Specifically, the a priori power for the paired t-tests comparing preoperative and postoperative measurements for both flaccid dorsal length and midshaft girth was below 0.15, which is considered low. Erect penis measurements were not recorded.

**Table 1:** Pre- and postoperative data.

Subject	Pre-op date	Pre-op penile measurements (cm)		Post-op date	Post-op penile measurements (cm)	
		Flaccid dorsal length	Flaccid midshaft girth		Flaccid dorsal length	Flaccid midshaft girth
Case 1	5/13/2019	6.250	8.750	5/17/2019	11.875	11.875
Case 2	2/9/2021	7.500	9.375	2/15/2021	10.625	14.375
Case 3	9/23/2021	10.000	10.625	9/27/2021	13.750	13.750
Case 4	11/15/2022	7.500	9.375	11/18/2022	12.500	12.500

Mean flaccid dorsal length increase = +4.375 cm; *p* value = 0.005.

Mean flaccid midshaft girth increase = +3.595 cm; *p* value = 0.005.

The mean sub-score values on the IIEF for erectile function, orgasmic function, sexual desire, intercourse satisfaction, and overall satisfaction were 27.75, 5.00, 8.25, 13.00, and 8.25, respectively (Table 2). The lower sub-score for orgasmic function is linked to the responses to question 9 regarding the frequency of ejaculation during sexual activity, where three patients reported “Almost never or never” and one reported “A few times”. The composite scores suggest a high level of patient satisfaction in terms of erectile function, sexual desire, and intercourse, with a moderate satisfaction concerning orgasmic function.

**Table 2:** IIEF questionnaire data.

Subject	Date collected	Erectile function	Orgasmic function	Sexual desire	Intercourse satisfaction	Overall satisfaction
Case 1	12/21/23	27	5	10	12	8
Case 2	12/21/23	29	5	7	15	10
Case 3	12/21/23	28	6	7	14	8
Case 4	12/21/23	27	4	9	11	7
Mean	N/A	27.75	5.00	8.25	13.00	8.25

No long-term adverse effects were reported among the participants in follow-up as long as 55 months. Moreover, none required additional ED treatments, including oral PDE-5i, post-surgery. At the time of this study’s publication, all patients reported improved erectile function, including the ability for penetrative sex, achieving and maintaining erections, and reaching orgasm.

## Discussion

This study evaluated the outcomes of Himplant^®^ placement in four post-RP men desiring penile enhancement and suffering from refractory ED. The primary goal was to cosmetically enhance the penis by increasing flaccid length and girth, but our findings suggest that Himplant^®^ placement may also improve erectile function. Notably, postoperative erectile function IIEF scores in these four patients exceeded the average scores reported for ED and no patient required long-term oral PDE-5i therapy, indicating an absence of ED following the Himplant^®^ procedure [24, 25].

As anticipated following RP, patients reported low ejaculatory function due to anejaculation; however, other domains of sexual health, including desire, intercourse satisfaction, and overall satisfaction, aligned with scores seen in healthy individuals without ED. Notably, no complications such as seroma, erosion, or infection associated with Himplant^®^ occurred and no further interventions were necessary for up to 55 months postoperatively.

The Himplant^®^ has been shown to enhance flaccid penile dimensions, potentially boosting self-esteem, and our study provides initial evidence of its ability to improve erectile function in men with mild-to-moderate post-RP ED. We hypothesize that the implant’s rigidity can potentially facilitate penetration independently of arousal-induced tumescence, while sexual activity may encourage cavernosal filling, thereby improving tumescence. This enhancement in erectile function appears to contribute significantly to high postoperative satisfaction with sexual intercourse, overall satisfaction, and sexual desire.

Strengths of this study include careful patient selection, the use of the IIEF for a comprehensive evaluation of erectile function and sexual satisfaction, an extensive follow-up period, and the achievement of favorable functional and cosmetic outcomes as perceived by the patients. However, the limitations are notable, including reduced statistical significance owing to the small cohort, limited generalizability, and the absence of erect penile measurements.

## Conclusion

This study introduces Himplant^®^ as a potential treatment option for men who are dissatisfied with the esthetic appearance of their penis and have mild-to-moderate post-RP ED. Based on this small case series, Himplant^®^ was shown to be effective for four patients in providing penile enhancement and improving post-RP ED characteristics without any long-term complications. Further multicenter studies with larger cohorts are necessary to validate the efficacy and durability of the results.

## Data Availability

All data generated or analyzed during this study are included in this published study and its supplementary information files.
